# Green space justice amid COVID-19: Unequal access to public green space across American neighborhoods

**DOI:** 10.3389/fpubh.2023.1055720

**Published:** 2023-02-02

**Authors:** Shuqi Gao, Wei Zhai, Xinyu Fu

**Affiliations:** ^1^School of Architecture, Southeast University, Nanjing, Jiangsu, China; ^2^School of Architecture and Planning, The University of Texas at San Antonio, San Antonio, TX, United States; ^3^Environmental Planning Programme, University of Waikato, Hamilton, New Zealand

**Keywords:** COVID-19, green space justice, stay-at-home order, mobile phone data, neighborhood analysis

## Abstract

Countries around the world have resorted to issuing stay-at-home orders to slow viral transmission since the COVID-19 pandemic. During the lockdown, access to public park plays a central role in the public health of surrounding communities. However, we know little about how such an unprecedented policy may exacerbate the preexisting unequal access to green space (i.e., green space justice). To address this research void, we used difference-in-difference models to examine socioeconomic disparities, urban-rural disparities, and mobility disparities in terms of public park access in the United States. Our national analysis using the weekly mobile phone movement data robustly suggests the following three key findings during COVID-19: (1) The elderly, non-college-educated people, poor people, and blacks are less likely to visit public parks frequently, while unemployed people appear to be the opposite. (2) Compared to rural areas, populations in urban neighborhoods appear to visit public parks more frequently and they generally go to larger parks to minimize the risk of infection. (3) Populations in neighborhoods with higher private vehicle ownership or those with a higher density of transit stops would more frequently visit and travel a longer distance to public parks during the stay-at-home order. Our results imply that conventional inequality in green space access may still exist and even become worse during COVID-19, which could negatively impact people's health during isolation. We suggest that special attention should be paid to park-poor neighborhoods during the pandemic and in the post-pandemic recovery phase.

## 1. Introduction

Since the outbreak of COVID-19, public health entities have repeatedly underscored the importance of practicing social distancing and staying at home to minimize interpersonal contact. Many restriction measures such as border closure, indoor gathering limitation, social distancing, and stay-at-home orders have been adopted around the world to slow viral transmission, to relieve the pressure on health care systems, and, simply, to prevent excess deaths (1). These restrictions can adversely affect people's wellbeing due to isolation and inadequate physical activities, thereby further reducing people's ability to combat the virus (2). Local streets, public parks, trails, etc., are the main places where outdoor activities take place (3). Therefore, public park visitations, including forest excursions, increased significantly during the restricted period in response to the policy (4). Public parks can not only serve as a substitute and shelter for the majority of the population during a sustained pandemic, as long as visitors take a more isolated path in parks (5), but also enhance longstanding resilience because of the positive impacts on people's mental and physical health (6, 7).

Improving access to public parks is a critical strategy with a high return on investment, especially for socially disadvantaged groups, who can benefit more from green space (8). However, socially and economically disadvantaged populations, such as low-income people (9), ethnic minorities (10), less-educated people (11), immigrants (12), and the elderly (13) are all well-documented to have less access to green space in terms of the size, amenities, maintenance standards and security levels (14). Additionally, the existing research on green space justice was largely focused on the urban context, while the urban-rural differences with respect to green space access can vary substantially (15). Moreover, insufficient mobility resources may further exacerbate inequality. In the UK, people returning to work are more likely to drive a private car than taking public transport for infection concerns (16). Nevertheless, people without a private vehicle still might not take public transit because many countries have restricted the operation of public transit for work or non-essential trips during the pandemic (3).

The lockdown during the pandemic would restrict people's mobility, especially for the marginalized populations who are primarily dependent on public transit. The pandemic thus might further exacerbate inequalities in green space access for people who cannot visit any park within walking distance. However, the impact of COVID-19 on such inequalities is yet understudied. To address this research gap, we analyzed the visitations to public parks from all neighborhoods in the contiguous United States (US) to answer the following three research questions: (1) whether disadvantaged neighborhoods with varying socioeconomic characteristics have sufficient access to public parks during COVID-19? (2) Whether neighborhoods in rural areas have sufficient access to public parks during COVID-19? (3) Whether mobility-poor neighborhoods have sufficient access to public parks during COVID-19?

Hence, this study first comprehensively explores the nationwide inequality in green space during the pandemic at the neighborhood level. Also, this work employed the multiple-source data to examine different types of inequality in the United States. The rest of this article proceeds as follows. We first review the existing relevant literature to provide a research contextual backdrop. We then present the data for this research and specify the methods used for the analysis. Finally, we present the findings and conclude with policy implications and future research.

## 2. Literature review

### 2.1. Social disparity and green space access during COVID-19

As a form of public assets, public parks are expected to be distributed equally across the neighborhoods (17, 18). However, the existing studies in environmental justice suggest that green space is not equally accessed due to socioeconomic disparities of neighborhoods (10, 19, 20).

Specifically, age is a determining factor that is pertaining to people's access to public parks during the pandemic. The elderly people have less access to public parks because of the inconvenience of mobility and need to be taken care of Iraegui et al. (13). Gated public parks for preventing antisocial activity unintentionally meant that the disabled elderly people could not access such spaces by themselves. Knowles and Hanson (21) found that the stay-at-home order confined older people to stay in indoor spaces, with fewer chances to go outside and visit public parks. In addition, Chiou and Tucker (22) found a similar finding that during COVID-19, neighborhoods with more older people have a high proportion of stay-at-home residents. Likewise, Dasgupta et al. (23) found that communities, with high compliance of social distancing during the pandemic, had 8.2% fewer youth and 7.4% more elderly.

Low-income neighborhoods lack access to high-quality public parks, especially for newly constructed low-socioeconomic communities (9). During a pandemic, poor people are more likely to visit small and congested green spaces, which are not suitable for physical exercise and risky for viral transmission (18). Zhai et al. (4) also observed that residents in low-income counties have fewer visitations to public parks than those in high-income counties in the US during the early outbreak of COVID-19.

Most of the ethnic minorities have lower socioeconomic status with less wage (24, 25), less car ownership (17, 26), and longer working time (27), which make them difficult to access public parks in longer distances. Also, ethnic minorities are more likely to face discrimination by visitors, police, and staff in green spaces (10, 28). By conducting a survey in New York City, Lopez et al. (29) found that public park use was lower for Hispanic communities, and the importance of public park for health was perceived as lower for black respondents during COVID-19. However, whether ethnic minorities across the US face insufficient access to public park have not be empirically evidenced.

Employment status is also determining people's access to public parks, especially considering that recent empirical studies have explored the heterogeneous impacts of governmental interventions on different occupations (30, 31), making a growing number of unemployed during the pandemic. Coombes et al. (32) adopted the employment rate as one the demographic measures for public park access. When examining the association between greenspace, urbanity, and human health in England, Mitchell and Popham (33) typically considered employment deprivation for statistical model controlling.

Inequalities with respect to education have been proved to be associated with green space access by the existing literature (34–36). Many case studies in Europe and North America have shown that access to either private green space or public green space is largely determined by education level (37–39). Moreover, Cole et al. (11) observed that the quality of green space is lower in less educated neighborhoods. Notably, well-educated people are more likely to trust science and comply with stay-at-home order (22), thereby actively reducing visitations to green spaces during the pandemic.

To sum up, the existing studies suggest that neighborhoods with varying median age (13), income (4), ethnic minorities (10), unemployment rate (33), and education level (11) may have heterogeneous access to public park. However, the research on the association between COVID-19 and public park access, as of spring 2021, is still limited because previous findings may not include the pandemic context. Specifically, previous studies mainly rely on cross-sectional survey data on routine days to examine the association between social disparity and public park access. However, few of the existing studies are in the context of COVID-19, regarding that due to the dynamic change of COVID-19 and unprecedent stay-at-home order, people's behaviors can be totally different from previous patterns (40). That is, the existing knowledge may not hold ground in many ways during such a pandemic, necessitating a comprehensive exploit on the public park access with the dynamic data.

### 2.2. Urban–rural disparity and green space access during COVID-19

Despite the primary focus on public park access in urban areas, rural areas should not be overlooked. The existing literature supports that people living in rural areas tend to have insufficient mobility (15) and low-quality park facilities (41), so that people in rural areas visit green space much less frequently (42). Wen et al. (15) found that the median distance to the near public parks for rural neighborhoods was 10 times that for neighborhoods in principle urban centers in the US. Likewise, in Europe, Wolff et al. (43) found that the average proximity to public park for urban residents is 13 times larger than that for rural residents. Maas et al. (8) and Mitchell and Popham (33) both found that the association between green space and health varied is determined by the degree of urbanity in an area. Richardson et al. (44) found that the effects of green space on residents' health outcomes may vary from the rural area to urban core because the role of green space is more impactful in the context of urban environments in contrast to rural environments. However, Zasada et al. (45) argue that increasing population density, the insufficient availability of green space, and the overuse of public parks can collectively lead to the decreasing attractiveness of recreational possibilities for urban residents. Mitchell and Popham (33) also argue that suburban and rural residents generally have their domestic gardens so that they have limited demand for public parks as compared to urban residents, rather than that they cannot access public parks.

Therefore, the impact of urban–rural disparity on public park access is still inconclusive when researchers use data on normal days, which could be more complicated during COVID-19, because Mueller et al. (46) found that the situation of the pandemic in rural areas have been dreadful, with significant negative influences on people's travel behavior, life satisfaction, and overall health. Rice et al. (47) found that, since the outbreak of COVID-19, the outdoor recreation activities and distance traveled have declined significantly more among urban residents than rural residents by conducting an online survey. Although urban-rural disparity has drawn widespread attention during COVID-19, the existing literature has not explored whether there is an association between the urban-rural disparity and public park access. However, addressing such question is essentially important for policymakers to identify and enhance park-poor neighborhoods from a geographic perspective, especially during the pandemic.

### 2.3. Mobility disparity and green space access during COVID-19

Mobility resource is another contributing factor for green space access. Nissen et al. (48) suggested that untangling the relation between mobility and green space can help enhance the wellbeing of people. Wendel et al. (49) found that public transit is the most frequent transport method for people to access green space, followed by private vehicles. Likewise, Fan et al. (50) demonstrated that the average travel time to public parks can be reduced either by improved public transport systems or higher availability of private vehicles. Haslauer et al. (51) considered the access to public transit as the main factor of green space availability. Europe (52) suggested that the 300-m buffer was chosen for measuring the public park access because 300 m represent a 5-min walking distance to the nearest transit stop, which ensures access to parks for people without a car. However, the closest park is not always the most visited. This is particularly true in Western car-oriented countries because public parks spreading over comparatively large areas is highly associated with mobility supplies (15).

Therefore, public transport and private vehicles have been widely acknowledged to impact public park access. However, during the pandemic, transit stops and vehicles are considered as high-risk environments due to the crowded environment, the plenty of surfaces that help spread the virus, and the insufficient testing of passengers (53). To this end, Zhang et al. (54) documented notable modal shifts away from public transit usage because over 60% of survey respondents agree that the car dependence of passengers may increase because of adverse response to crowded public transit environment during the pandemic. Wilbur et al. (55) found a drop of transit ridership in Tennessee, USA due to COVID-19, which may keep people away from green spaces. In New York City, Teixeira and Lopes (56) also found that some transit users changed mode to the bike sharing service. Since the outbreak of COVID-19, even though we know that the mode shift occurs, how the public transport resource and car dependence have impacted neighborhood-level access to nature parks has not been empirically understood, complicating the decision-making process of bridging the gap of green space inequality from a transport perspective.

## 3. Data

### 3.1. Data source

In this study, we chose all the census tracts in the contiguous US to be our study area. After excluding the ones with missing data, a total of 69,867 census tracts were included for the subsequent analysis. Then, we first retrieved weekly mobile phone movement data and visitor insights data for physical places from SafeGraph (57) (https://www.safegraph.com/). Our dataset ranges from January to May in both 2019 and 2020. Based on the Points of Interest (POI) category, we extracted 90,013 urban-park POIs within the contiguous US. A POI is a specific point location that someone may find useful or interesting. Thereafter, we connected all the urban-park POIs with their origin neighborhoods to quantify the measures of access to public parks for each neighborhood, including the average distance people traveled to public parks, the average size of the public parks people visited, and the proportion of people who have visited public parks. These three metrics are derived from the existing literature pertaining to public park access (20, 58). The detailed description of such dataset and the metrics can be found in Supplementary material.

Second, to explore the effects of socioeconomic and demographic characteristics on public park access, we retrieved the 2018 American Community Survey (ACS) database (https://www.census.gov/programs-surveys/acs/data.html) to construct estimates of the poverty rate, the percentage of non-college-educated people, the percentage of elderly people (age 65+), the unemployment rate (i.e., the percentage of people who are not employed before the pandemic, excluding retirees), the percentage of blacks for each neighborhood. Note that we mainly consider the blacks in this study because the literature indicates that blacks are the most vulnerable to COVID-19 transmission (59–61).

Third, to understand the urban-rural disparities, we employed the urban-rural classification scheme developed by the National Center for Health Statistics (62) for all US counties (https://www.cdc.gov/nchs/data_access/urban_rural.htm). Specifically, six levels (Large Central Metropolitan, Large Fringe Metropolitan, Medium Metropolitan, Small Metropolitan, Micropolitan, Noncore Area) are categorized in the classification scheme, which is determined by the population size of the corresponding county.

Fourth, in terms of the mobility data, we collected the locations of transit stops across all the neighborhoods from the General Transit Feed Specification (63) (https://gtfs.org/). By overlaying the bus stops with census tract by ArcGIS 10.6, we can calculate the density of transit stops for all neighborhoods. In addition, the average number of private vehicles for each household can also be collected from ACS database.

We assume that the public social-distancing behaviors are guided and influenced by the stay-at-home order. To this end, we collected statewide stay-at-home orders from Mervosh et al. (64). Specifically, 43 states had issued stay-at-home orders to encourage residents to shelter in place. However, some counties had also issued a more stringent local order than the state. For instance, the Florida governor did not issue the state-level stay-at-home order until April 1^st^, while the majority of Florida counties had already put local directives in place by March 25th. Hence, we also collected a county-level stay-at-home order from Keystone Strategy (65). Specifically, 592 out of 3,142 counties had issued a county-level order. We later combined the county-level and state-level orders to determine whether a county was under a stay-at-home order based on the earlier order.

### 3.2. Descriptive statistics

**Figure 1:**
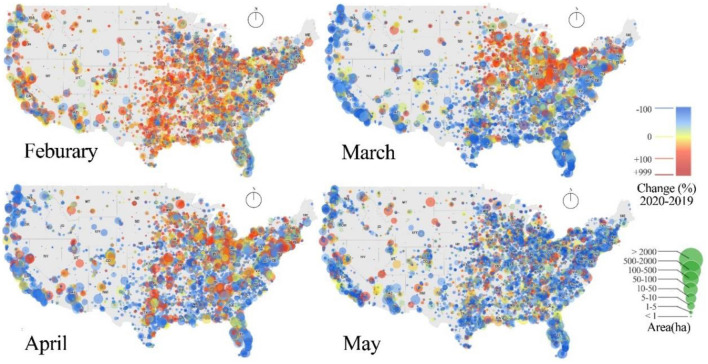
The change in the number of public park visitors between 2019 and 2020.

Table 1 provides summary statistics of the key variables. Note that the socioeconomic variables, urban-rural variables, and mobility resource variables are all cross-sectional data while the remaining variables are panel data. Since visitation to public parks exhibits varying seasonal patterns (66), showing the variations solely in 2020 cannot explicitly reflect people's behavior changes due to COVID-19. Figure 1 illustrates the interannual change of visitation to public parks based on the data of 2019 and 2020. In the US, the first case of COVID-19 was reported in January 2020, but a national emergency was not declared until March 13^th^, 2020. Therefore, in January, it is not surprising that the number of visitors in 2020 even increased compared to that in 2019. In February 2020, for over 60% of parks, the number of visitors has increased by at least 5% compared to that in February 2019. However, with the pandemic on a rampage in March and April, the total number of visitors had thus fallen by 48%, and the number of visitors in over two-third of public parks significantly declined, particularly in the West Coast, East Coast, and the Southern US. Meanwhile, interestingly, the Midwestern US still had a significant increase. Starting in May, the visitations to most parks have clearly declined across the country.

**Table 1:** Descriptive statistics of all variables for all census tracts.

	**Description**	**Type**	***N***	**Mean**	**Std. Dev**	**Min**	**Max**
**Dependent variable**							
*Year 2020 (January–May)*							
Distance (km)	The average distance that people travel to public parks in 2020.	Weekly	1,108,086	19.78	31.48	0.12	427
Size (km^2^)	The average area of public parks that people travel to in 2020.	Weekly	1,108,086	0.01	0.04	0.001	1.15
Visiting public parks	The proportion of a neighborhood that people visit public parks in 2020.	Weekly	1,108,086	0.14	0.11	0	1
*Year 2019 (January–May)*							
Distance (km)	The average distance that people travel to the public parks in 2019.	Weekly	1,108,086	23.50	32.11	0.12	538
Size (km^2^)	The average area of public parks that people travel to in 2019.	Weekly	1,108,086	0.01	0.03	0.001	1.15
Visiting Public parks	The proportion of a neighborhood that people visit public parks in 2019.	Weekly	1,108,086	0.06	0.04	0	1
**Independent variables**							
*Socioeconomic and demographic characteristics*							
Elderly	The proportion of people who are older than 60 years old	Cross-sectional	69,867	0.16	0.08	0	1
No college degree	The proportion of non-college-educated people	Cross-sectional	69,867	0.79	0.14	0	1
Poverty	The proportion of people in poverty status	Cross-sectional	69,867	0.15	0.11	0	1
Black	The proportion of Black people in the neighborhood.	Cross-sectional	69,867	0.13	0.23	0	1
Unemployment	The proportion of unemployed people	Cross-sectional	69,867	0.06	0.04	0	0.5
*Urban-rural variables*							
Large central metro	Neighborhoods in metropolitan statistical area (MSA) of 1 million population with three conditions^a^.	Cross-sectional	21,920				
Large fringe metro	Neighborhoods in MSAs of 1 million or more population that do not qualify as the large central metro.	Cross-sectional	15,445				
Medium metro	Neighborhoods in MSA of 250,000–999,999 population	Cross-sectional	14,289				
Small metro	Neighborhoods in MSAs of < 250,000 population	Cross-sectional	6,307				
Micropolitan	Neighborhoods in micropolitan statistical areas	Cross-sectional	6,349				
Noncore area	Neighborhoods not in a micropolitan statistical area	Cross-sectional	5,113				
*Mobility variables*							
No vehicle available	The proportion of household that has no vehicle	Cross-sectional	69,867	0.09	0.12	0	1
One vehicle available	The proportion of household that has one vehicle	Cross-sectional	69,867	0.33	0.12	0	1
Two vehicles available	The proportion of household that has two vehicles	Cross-sectional	69,867	0.37	0.11	0	1
Three vehicles available	The proportion of household that has three vehicles	Cross-sectional	69,867	0.14	0.07	0	1
Four or more vehicles available	The proportion of household that has four or more vehicles	Cross-sectional	69,867	0.07	0.05	0	1
Density Of Transit Stops	Number of transit stops per square mile	Cross-sectional	69,867	11.26	24.06	0	484.38
*Stay-at-home order*							
Under order	The census tract is under the stay-at-home order	Weekly	490,353				
Not under order	The census tract is not under the stay-at-home order	Weekly	618,633				

^a^(1) Contain the entire population of the largest principal city of the MSA, or (2) are completely contained in the largest principal city of the MSA, or (3) contain at least 250,000 residents of any principal city of the MSA. A metropolitan statistical area (MSA) is a geographical region with a relatively high population density at its core and close economic ties throughout the area.

## 4. Method

### 4.1. Examining socioeconomic disparities

The difference-in-differences (DID) method is a quasi-experimental approach that compares the changes in outcomes over time between the treatment group and the comparison group. We first used the DID estimation method to compare neighborhoods with varying socioeconomic characteristics before and after the issuance of stay-at-home order.


(1) $$\begin{array}{l} Metrics_{nit}=\alpha_{1}PostOrder_{it}+\alpha_{2}PostOrder_{it}\times Elderly\\ \;+\alpha_{3}PostOrder_{it}\times NoDegree+\alpha_{4}PostOrder_{it}\\ \;\times Poverty+\alpha_{5}PostOrder_{it}\times Unemployment\\ \;+\alpha_{6}PostOrder_{it}\times Black+\gamma_{i}+\delta_{t}\;+\;u_{it} \end{array}$$

For *Metrics*_*nit*_, it represents *n*-th metrics for access to public parks, including distance traveled to public parks, the average area of public parks that people traveled to, and the percentage of people visited public parks in the census tract *i* on week *t*. We define that calls for stay-at-home represents the outbreak of each county so that we use the issuance date as the cutoff to determine the “treatment” period. Coefficient α_1_ captures the effects of the stay-at-home order on different metrics. Note that, throughout all the models, we define *PostOrder*_*it*_ = 1 when the stay-at-home has been in place in the census tract *i*; otherwise, it is zero. Coefficient α_2_, α_3_, α_4_, α_5_, and α_6_ represents the effects of the proportion of elderly people, the proportion of non-college-educated people, the poverty rate, the unemployment rate, the proportion of blacks on the access metrics, respectively.

In particular, γ_*i*_ represents census tract-specific dummy variables that take a value of 1 for census tract *i* and a value of zero for other census tracts. These fixed effects can guarantee that census tract-specific factors, which are constant over time are controlled for during the investigation. In addition, δ_*t*_ represents week-specific dummy variables that take a value of 1 for week *t* and a value of zero for other days. These fixed effects guarantee that week-specific factors, which are common across neighborhoods, are controlled for during the investigation. Finally, *u*_*it*_ represents residuals.

To avoid bias due to the seasonality of visitation to public parks, we conducted the analysis by comparing the changes between 2019 and 2020, as indicated in Equation (A2).

### 4.2. Examining urban-rural disparities

We then examined the unequal access to public parks in urban and rural areas by using the following DID model:


(2) $$\begin{array}{l} Metrics_{nit}=\beta_{1}PostOrder_{it}+\beta_{2}PostOrder_{it}\times LargeCentral\\ \;+\beta_{3}PostOrder_{it}\times LargeFringe+\beta_{4}PostOrder_{it}\\ \;\times MediumMetro+\beta_{5}PostOrder_{it}\times SmallMetro\\ \;+\beta_{6}PostOrder_{it}\times Micropolitan+\gamma_{i}+\delta_{t}\;+\;u_{it} \end{array}$$

Coefficient β_1_ captures the effects the stay-at-home order on different metrics of access to public parks. Coefficient β_2_, β_3_, β_4_, β_5_, and β_6_ captures the additional effects of the large central metro area, large fringe metro area, medium metro area, small metro area, and micropolitan on the access metrics, respectively. Note that the variable “Noncore Area,” which represents the census tracts in rural areas, is the reference variable so that it is not indicated in the model. The interpretations for γ_*i*_ and δ_*t*_ are same as that in Equation (1). Again, to avoid the bias of seasonal effects, we robustly examine urban-rural disparities using the data of 2019 and that of 2020, as shown in Equation (A3).

### 4.3. Examining mobility disparities

Lastly, the formal investigation on mobility resources is also accomplished by using a DID model, where weekly changes of access to public parks are regressed on the stay-at-home order and availability of vehicles.


(3) $$\begin{array}{l} Metrics_{nit}=\theta_{1}PostOrder_{it}+\theta_{2}PostOrder_{it}\times OneVehicle\\ \;+\theta_{3}PostOrder_{it}\times TwoVehicle+\theta_{4}PostOrder_{it}\\ \;\times ThreeVehicle+\theta_{5}PostOrder_{it}\times FourMoreVehicle\\ \;+\theta_{6}PostOrder_{it}\times Transit+\gamma_{i}+\delta_{t}\;+\;u_{it} \end{array}$$

Coefficient θ_1_ captures the effects the stay-at-home order on different metrics of access to public parks. Coefficient θ_2_, θ_3_, θ_4_, θ_5_, and θ_6_ captures the additional effects of availability of one vehicle, availability of two vehicles, availability of three vehicles, availability of four or more vehicles, and the density of transit stops, respectively. Note that the variable “No Vehicle Available” is the reference variable so that it is not indicated in the model. The interpretations for γ_*i*_ and δ_*t*_ are same as that in Equation (1). Once again, we also robustly examine urban-rural disparities using the data of 2019 and that of 2020, as indicated in Equation (A4).

## 5. Results

### 5.1. Socioeconomic inequality

Table 2 presents the model results of the effect of government order on the average travel distance to parks, the average size of accessible parks, and the percentage of visitation to public parks. Specifically, column (1), (3), and (5) show the raw effects of stay-at-home order on the key dependent variables. Column (1) and (3) generally indicate that people may visit public parks at a longer distance but with a larger area. It might be because under the stay-at-home policy people were more likely to visit public parks with low population density for a lower risk of infection. Column (5) implies that more people would go to public parks during the pandemic, confirming our primary assumption based on the existing studies [e.g., (4)].

**Table 2:** Before and after stay-at-home order.

	**Distance**		**Size**		**Percentage of visitation**	

	**(1)**	**(2)**	**(3)**	**(4)**	**(5)**	**(6)**
Order	0.001^***^ (0.0003)	0.030^***^ (0.012)	0.003^***^ (0.0003)	0.045^***^ (0.011)	0.076^***^ (0.002)	0.946^***^ (0.008)
Order × elderly		−0.018^**^ (0.002)		−0.005^***^ (0.0017)		−0.005^***^ (0.001)
Order × no college degree		−0.038^***^ (0.015)		−0.054^***^ (0.013)		−1.104^***^ (0.010)
Order × poverty		−0.011^***^ (0.003)		−0.010^***^ (0.002)		−0.070^***^ (0.002)
Order × unemployment		0.009^***^ (0.002)		0.008^***^ (0.002)		0.045^***^ (0.001)
Order × black		0.018^***^ (0.002)		−0.004^***^ (0.002)		−0.023^***^ (0.001)
Census tract fixed effects	Yes	Yes	Yes	Yes	Yes	Yes
Date fixed effects	Yes	Yes	Yes	Yes	Yes	Yes
Observations	1,073,800	1,073,800	1,073,800	1,073,800	1,073,800	1,073,800
*R*-squared	0.37	0.37	0.50	0.50	0.70	0.71

p < 0.01^***^; p < 0.05^**^.

Column (2) indicates that the elderly, non-college-educated people, and people in poverty status would generally visit parks near their homes. It might be due to their low mobility as many of these people have limited transportation options. The pandemic lockdown further exacerbated such green space inequalities for these marginalized groups when the public transportation systems were either closed or restricted. However, blacks appeared to travel longer for park visits. It might be because blacks have long suffered from spatial inequality in terms of limited access to free public parks that, in turn, forced them to travel longer distances for parks. For unemployed people, they could travel a long distance to a park without extra concern about the commuting time because of no work obligations.

In column (4), the decrease of the average area further confirms our explanation for the decreasing travel distance of the elderly, non-college-educated people, and poor people, because they mainly prefer nearby small parks than distant large parks. Moreover, unemployed people are more likely to visit larger parks since they could travel a longer distance. However, even though blacks could travel a longer distance, they still could go to a smaller park. It could be because some large parks, such as state parks or national parks, require parking fee or entrance fee, while smaller park is free to access and park. It could also be that blacks have concern that they would be discriminated in large parks where the white people are interested in Gobster (28). That is, blacks might not choose to visit a larger but farther park for a lower risk of infection because they generally show less trust in science during this particular pandemic (40, 67).

Column (6) indicates that the elderly and non-college-educated people may reduce their visitations to public parks during the lockdown. The elderly is knowingly the most vulnerable population group amid this pandemic so that they would be more cautious about potentially contracting the virus. Furthermore, non-college-educated and low-income people are less likely to have stable work, which could be worsened by COVID-19, leading them to have less free time for leisure. Further, it has been widely acknowledged that low-income people might not be able to afford admission fees of public parks so that they would travel long distances to visit free parks. Likewise, blacks would also have less chance to visit parks because they are more likely to be in poverty status and usually suffer from poor transport mobility. Interestingly, unemployed people appeared to increase their visits to public parks during the lockdown. This might be explained by the extra free time they had without work obligations so that they would be more willing to visit farther public parks when the majority of public spaces had been restricted.

Supplementary Table 1 shows the effects of socioeconomic and demographic variables using data in 2019 and 2020 to reduce the bias of the seasonal difference. The results still support our earlier findings from Table 2, thereby further demonstrating the robustness of our analysis.

### 5.2. Urban-rural inequality

Table 3 indicates the results from the analysis of examining the comparative effects of the urban and rural areas on people's access to public parks. Column (1) shows that people living in metro areas would travel a longer distance to public parks than people in non-core areas (i.e., rural areas in this study) after the issuance of stay-at-home orders. As confirmed in column (2), it could be because people in urban areas would be more likely to visit larger parks with more space to exercise public health measure and thus face a lower risk of infection, despite that the coefficient estimates are not statistically significant for small metro areas and micropolitan areas. As shown in column (3), people in the metro areas would be more likely to visit public parks compared to the rural areas. It is worth noting that with the area being more populated, the coefficients for increased distance and increased percentage of visitation would be greater, suggesting that green space inequality was worsening in less populated areas. Again, we examined the urban-rural disparity using the data in 2019 and 2020 and our findings are still robust (Supplementary Table 2).

**Table 3:** Urban–rural disparities before and after stay-at-home order.

	**(1) Distance**	**(2) Size**	**(3) Percentage of visitation**
Order	−0.126^***^ (0.008)	−0.0122^*^ (0.007)	0.003^***^ (0.001)
Order × large central Metro	0.174^***^ (0,008)	0.024^***^ (0.007)	0.175^***^ (0.005)
Order × large fringe metro	0.147^***^ (0.008)	0.027^***^ (0.007)	0.044^**^ (0.005)
Order × medium metro	0.114^***^ (0.008)	0.021^***^ (0.007)	0.040^***^ (0.005)
Order × small metro	0.058^***^ (0.009)	−0.007 (0.008)	0.011^*^ (0.006)
Order × micropolitan	0.051^***^ (0.009)	0.003 (0.008)	0.004 (0.006)
Census tract fixed effects	Yes	Yes	Yes
Date fixed effects	Yes	Yes	Yes
Observations	1073,800	1073,800	1073,800
*R*-squared	0.37	0.50	0.70

p < 0.01^***^; p < 0.05^**^; p < 0.1^*^.

### 5.3. Mobility inequality

Table 4 shows the model results about the effects of mobility on green space access. Surprisingly, column (1) indicates that households with more vehicles would increase the average distance traveled to public parks after the stay-at-home order, except that the coefficient estimate of Four Or More Vehicles Available is not statistically significant. Furthermore, it also suggests that when the density of transit stops increases people in the neighborhoods would travel a long distance to visit public parks. Column (2) indicates that the availability of vehicles might not impact people's choice for the size of the parks. However, a higher density of transit stops would enable people to visit a large public park, again, where the infection risk is lower. Column (3) shows that the increase in household car ownership would in turn increase the visitation to public parks even during the pandemic. Similarly, the increasing provision of transit stops would also lead to more park visits.

**Table 4:** Mobility disparities before and after stay-at-home order.

	**(1) Distance**	**(2) Size**	**(3) Percentage of visitation**
Order	0.004^**^ (0.002)	0.005^**^ (0.002)	0.074^***^ (0.002)
Order × one vehicle available	0.018^***^ (0.003)	0.010 (0.008)	0.039^***^ (0.002)
Order × two vehicles available	0.010^***^ (0.002)	0.003 (0.002)	0.064^***^ (0.001)
Order × three vehicles available	0.017^***^ (0.003)	0.0002 (0.002)	0.069^***^ (0.001)
Order × four or more vehicles available	0.0003 (0.002)	−0.001 (0.002)	0.030^***^ (0.001)
Order × density of transit stops	0.015^***^ (0.002)	0.006^***^ (0.001)	0.051^***^ (0.001)
Census tract fixed effects	Yes	Yes	Yes
Date fixed effects	Yes	Yes	Yes
Observations	1,143,236	1,143,236	1,143,236
*R*-squared	0.37	0.50	0.70

p < 0.01^***^; p < 0.05^**^.

The results are generally consistent with the conventional wisdom that mobility-rich neighborhoods are more accessible to green spaces (68). Thus, mobility-poor neighborhoods should be given more attention during the pandemic, considering that they generally have limited access to public parks and could thus significantly suffer from the resulting social and physical isolation. Furthermore, choosing to drive to public parks during the pandemic has many benefits, especially driving alone or driving with household members, because people do not have to contact strangers as they would through transit. Again, the results are also validated by using the data of 2019 and 2020 (Supplementary Table 3).

## 6. Discussions

This research sheds light on landscape planning and public health management through the lens of green space justice during a global pandemic. Equal access to green spaces has long been advocated for just spatial planning, but the existing class division has seemed to widen the injustice gap with respect to public park access due to this pandemic. For instance, inequalities in park acreage and distance are evident across American communities. However, planning does not operate in a wonderland with utopian blueprints coming into fruition, while it indeed is as much as a political process as an economic and social one. The most challenging in practice is certainly balancing the competing interests among the different stakeholders such that the government has to acquire significant amounts of parkland, partner with various agencies, and require developers to include parkland in their subdivisions, whereas the developers aim to minimize such public good investments to maximize their profits (14).

Negotiations will follow and compromises will be made, but our results clearly show that there is still large room for improvement to close the gap of green space inequality in the US. Local governments could link their park systems to neighborhoods with similar socioeconomics and demographics, population, and mobility resources during the planning process to uncover some specific issues within local park systems, such as park deserts, and help them prioritize future investments. Lowering the admission fee and offering safe transports to public parks might also be more feasible for socially vulnerable communities during COVID-19 because these do not necessitate the acquisition of new parkland. We urge policymakers to integrate green space justice into the spatial organization of public parks because guaranteeing people's access to public parks has seldom to be a central component of the urban sustainability agenda (69, 70). Urban neoliberal policies have resulted in a global surge in the privatization of green space (71). While revenues from private business interests (e.g., cafés, stores) make park restoration and management financially sustainable, it occurs at the expense of public green space and the exclusion of disadvantaged groups, which is especially evident amid the pandemic.

Our results suggest that public health practitioners and researchers should pay more attention to park-poor neighborhoods, particularly the aging neighborhoods, low-income neighborhoods, black neighborhoods, and mobility-poor neighborhoods, during and after this pandemic. Access to public parks is essential for vulnerable populations because these socially marginalized groups of people would suffer even more, e.g., server anxiety, due to physical isolation (2). Regional agencies could also pay more attention to rural residents where access to public parks seem to not be adequate amid the pandemic. For neighborhoods with low availability of private vehicles, policymakers could offer interim mobility services. For instance, during the city lockdown, transit agencies could continue the operation of public transit that connects public parks and vulnerable neighborhoods, requiring public transit users to wear face coverings or masks and practice social distancing.

This study can be improved by addressing the following limitations. First, we only focused on the distributive justice of public parks. That is, subsequent studies could analyze procedural justice and interactional justice during COVID-19 using more evidence. Second, we adopted the mobile phone location data that may not exhaustively represent some underrepresented groups such as the elderly, poor people, and non-college-educated people, who might not own a smartphone or use it frequently. Future research should use some pilot study areas to further test the validity and robustness of this dataset. Third, due to the computation-intensive analysis of all neighborhoods in the contiguous US, spatial dependency cannot be considered in our regression analysis. Future studies could select a comparatively small study area to account for the spatial dependency in the analysis. Fourth, the composition of green space in non-urban areas is very complex, including natural grassland, marsh, etc. in addition to public parks, so that our analysis has not comprehensively considered these types of green space.

## 7. Conclusion

Our study contributes to understanding green space justice during the COVID-19 from the perspectives of socioeconomic and demographic inequalities, urban and rural inequalities, and mobility inequalities. The main strength of this study lies in the comprehensive examination of green space inequality during the pandemic. Another strength is that this research studied all the neighborhoods across United States. Our findings are threefold.

- After the stay-at-home is issued, the elderly, non-college-educated people, and poor people would be more likely to travel a less distance, visit relatively small public parks, and visit public parks less frequently. Blacks could also visit public parks less frequently during the stay-at-home order. Further, we found that unemployed people would increase their visits to public parks because they have more free time and do not have to work during the pandemic.
- Compared to rural areas, neighborhoods in urban areas show significant advantages in terms of visiting public parks more frequently and visit a large park to minimize infection risk.
- Mobility-rich neighborhoods may have better access to public parks, particularly for neighborhoods with more private vehicles available. Even though transit service may be beneficial to people's access to public parks, taking transit buses would further expose people to the virus. Regarding the potential health benefits of public green space, the unequal access to green space may exacerbate health inequalities, particularly during such an unprecedented pandemic. We argue that the government should devote special efforts to park-poor neighborhoods during and after the pandemic.

## Data availability statement

The original contributions presented in the study are included in the article/Supplementary material, further inquiries can be directed to the corresponding author.

## Author contributions

SG: conceptualization, writing—original draft preparation, review and editing, and visualization. WZ: methodology, data curation, writing—original draft preparation, and investigation. XF: review and editing, resources, validation, and data curation. All authors contributed critically to the manuscript and agreed to publication.
