# Supplementary material for: Green space justice amid COVID-19: Unequal access to public green space across American neighborhoods
Source: Front Public Health. 2023 Feb 2;11:1055720. doi: 10.3389/fpubh.2023.1055720 (PMC9932189; doi:10.3389/fpubh.2023.1055720)
Supplement: Supplementary file 1 [file Data_Sheet_1.docx]

***Supplementary Material***

1. **Supplementary Data**

We mainly adopted two pieces of dataset from the SaftGraph database: POI and weekly movement pattern. The POI dataset includes primary attributes such as location name, address, coordinates, category (e.g., public park, restaurant, shopping mall, school, etc.), and area of the POI footprint for over 60 million POIs across the United States. In this research, we only used the urban-park POIs and excluded the remaining data records in the dataset. The weekly movement data was collected using a set of GPS pings from 45 million encrypted mobile phones across the country. The common nighttime location (six-week period) of each mobile phone is determined to a Geohash-7 granularity (~153m x ~153m). Thereafter, we define this common nighttime location as the mobile phone user’s home. The opened data start from January 1, 2019 to December 31, 2020. By connecting the movement data and POIs, we can easily know where people visit, how often people visit, where they are living, where else they go, and more. More specifically, for each POI, we have a mapping of census tracts, where the mobile phone users’ homes are, to the number of visitors to the POI.

Based on the two datasets provided by SafeGraph, we then aggregate the devices at the census tract level and generate three metrics for access to public parks.

- **Mean Distance:** The average distance (in kilometers) traveled from the home census tract by the devices to public park POIs during each week (excluding any distances of 0).
- **Size**: The average area of public park POIs that people from the specified census tract have visited.
- **Percentage of Visitation:** We first obtain the count of unique mobile phone devices residing at a primary nighttime location in the specified census tract, which is estimated as the total mobile device users. We then estimate the percentage of people who have visited public parks in a week, given that we know how many devices have visited public park POIs.

1. **Supplementary Method: DID model based on year**

Difference-in-difference (DID) model is a classic quasi-experimental approach that applies panel data from treatment and control groups to obtain an appropriate counterfactual to estimate a causal effect. Therefore, DID is often employed to examine the effect of a specific intervention, such as the enactment of policy, or stay-at-home order in this research. Conventionally, DID is reflected as an interaction term between the time and treatment group dummy variables to compare the changes in outcomes over time between treatment group and control goup.
*Y= α_0_ + α_1_Time + α_2_ Treatment + α_3_Time* $\times$ *Treatment +u* (A1)

In Equation (1), coefficient *α_1_* represents the time trend; *α_2_* represents the difference between two groups pre-intervention; *α_3_* represents the DID estimator, indicating the differences in changes over time (as shown in **Supplementary Figure 1**). The assumption of applying DID model in this research is that of parallel trends, i.e., that without COVID-19 or stay-at-home order, access to public parks in census tracts with high and low factors would follow parallel trends.

Note that the research focus of our research is to understand whether the intervention or the pandemic has more or less impacts in unprivileged neighborhoods. Hence, we modified Equation (1) by replacing the variable *Time* with the variables of interests in this research. For example, when the variable of interest is the poverty rate, *α_3_* would represent the effects of the order on public park access in neighborhoods with higher poverty rate compared to that in neighborhoods with lower poverty rate. Regarding that the variables of interests in this research, such as socioeconomic variables, mobility resource variables, and urban-rural variables, are time invariant, the coefficient for the corresponding variable would be dismissed.


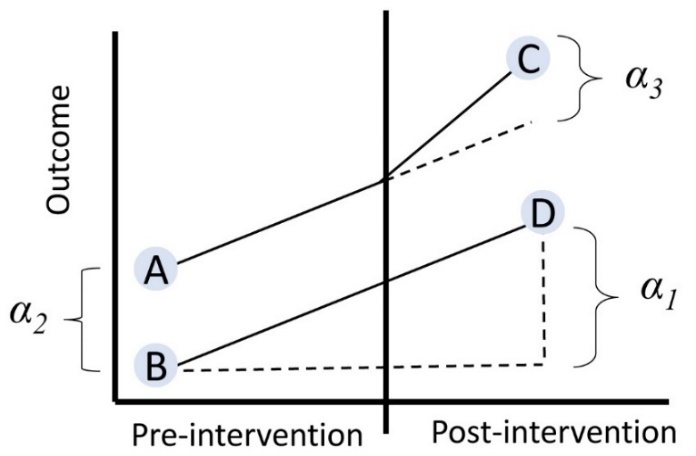


**Supplementary Figure 1**. Graphical illustration of the difference-in-difference estimation

To avoid the seasonal effects on people’s visitation to public parks, we used the data during the same period (January- May) of 2019 and 2020 to build the DID estimation model. That is to say, we assume that the year of 2020 as the ‘treatment’ period so that the variable *PostOrder* in Equation (1), (2), and (3) is replaced by *Year2020* in Equations (1), (2), and (3) below.

For the ease of presentation, the following variables and coefficients represent the same meaning in all the equations below. ${Metrics}_{nit}$ represents *n*-th metrics for access to public parks, including distance traveled to public parks, the average area of public parks that people traveled to, and the percentage of people visited public parks in the census tract *i* on week *t*. In addition, we define ${Year2020}_{it}$=1 when the year of date *t* is 2020; otherwise, it is zero. $u_{it}$ represents residuals. In particular, $\gamma_{i}$ represents census tract-specific dummy variables that take a value of 1 for census tract *i* and a value of zero for other census tracts. *δ_t_* represents week-specific dummy variables that take a value of 1 for week *t* and a value of zero for other weeks.

**1.1 Examining socioeconomic inequality**

Equation (2) aims to compare the effects of neighborhoods with varying socioeconomic characteristics on public park access in 2019 to that in 2020.

${Metrics}_{nit}=\alpha_{1}{Year2020}_{it}+\alpha_{2}{Year2020}_{it}\times Elderly+\alpha_{3}{Year2020}_{it}\times NoDegree+\alpha_{4}{Year2020}_{it}\times Poverty+\alpha_{5}{Year2020}_{it}\times Unemployment+\alpha_{6}{Year2020}_{it}\times Black+\gamma_{i}+\delta_{t} + u_{it}$ (A2)

Coefficient $\alpha_{1}$ captures the effects of year 2020 on different metrics compared to year 2019. Coefficient $\alpha_{2}$, $\alpha_{3}$, $\alpha_{4}$, $\alpha_{5}$, $\alpha_{6}$ represents the effects of the proportion of elderly people, the proportion of non-college-educated people, the poverty rate, the unemployment rate, the proportion of blacks on the access metrics, respectively. The results can be found in Supplementary Table 1. Note that column (1), (3), and (5) show the raw effects of year 2020 on the dependent variables compared to year 2019.

**Supplementary Table 1.** Social Disparity by Comparing Year 2019 and Year 2020

|  | Distance | | Size | | Percentage of Visitation | |
| --- | --- | --- | --- | --- | --- | --- |
|  | (1) | (2) | (3) | (4) | (5) | (6) |
| Year 2020 | 0.069***  （0.001） | 0.081***  (0.009) | 0.083***  (0.001) | 0.175***  （0.008） | 0.393***  (0.001) | 1.933***  (0.007) |
| Year 2020× Elderly |  | -0.020***  (0.001) |  | -0.004***  （0.001） |  | -0.036***  (0.001) |
| Year 2020×No college degree |  | -0.022***  (0.012) |  | -0.116***  (0.010) |  | -1.950***  (0.008) |
| Year 2020× Poverty |  | -0.006***  (0.002) |  | -0.009***  (0.002) |  | -0.130***  (0.001) |
| Year 2020× Unemployment |  | 0.008***  (0.002) |  | 0.007***  (0.002) |  | 0.054***  (0.001) |
| Year 2020× Black |  | 0.017***  (0.002) |  | -0.006***  (0.001) |  | -0.040***  (0.001) |
| Census tract fixed effects | Yes | Yes | Yes | Yes | Yes | Yes |
| Observations | 1,617,424 | 1,617,424 | 1,617,424 | 1,617,424 | 1,617,424 | 1,617,424 |
| R-squared | 0.37 | 0.37 | 0.48 | 0.48 | 0.66 | 0.68 |

p<0.01 ***; p<0.05 **; p<0.1 *

**1.2 Examining urban-rural inequality**

To further examine the urban-rural inequality, we adapt Equation (A2) to the following DID estimation.

${Metrics}_{nit}=\beta_{1}{Year2020}_{it}+\beta_{2}{Year2020}_{it}\times LargeCentral+\beta_{3}{Year2020}_{it}\times LargeFringe+\beta_{4}{Year2020}_{it}\times MediumMetro+\beta_{5}{Year2020}_{it}\times SmallMetro+\beta_{6}{Year2020}_{it}\times Micropolitan+\gamma_{i}+\delta_{t} + u_{it}$ (A3)

Coefficient $\beta_{1}$ captures the effects of year 2020 on different metrics. Coefficient $\beta_{2}$, $\beta_{3}$, $\beta_{4}$, $\beta_{5}$, $\beta_{6}$ captures the additional effects of the large central metro area, large fringe metro area, medium metro area, small metro area, and micropolitan on the access metrics, respectively.

**Supplementary Table 2.** Urban-rural Disparities by Comparing Year 2019 and Year 2020

|  | (1)  Distance | (2)  Size | (3)  Percentage of Visitation |
| --- | --- | --- | --- |
| Year 2020 | -0.186***  (0.005) | 0.045***  (0.003) | 0.113***  (0.003) |
| Year 2020× Large central metro | 0.132***  (0.005) | 0.034***  (0.004) | 0.486***  (0.004) |
| Year 2020× Large fringe metro | 0.106***  (0.005) | 0.081***  (0.005) | 0.257***  (0.004) |
| Year 2020× Medium metro | 0.073***  (0.006) | 0.030***  (0.005) | 0.233***  (0.004) |
| Year 2020× Small metro | 0.037***  (0.006) | 0.004  (0.006) | 0.169***  (0.005) |
| Year 2020× Micropolitan | 0.028***  (0.006) | 0.019***  (0.006) | 0.089***  (0.005) |
| Census tract fixed effects | Yes | Yes | Yes |
| Date fixed effects | Yes | Yes | Yes |
| Observations | 1,617,424 | 1,617,424 | 1,617,424 |
| R-squared | 0.37 | 0.48 | 0.68 |

p<0.01 ***; p<0.05 **; p<0.1 *

**1.3 Examining mobility inequality**

Equation (A4) aims to reflect the effects of mobility resources on public park access in 2020 compared to that in 2019.

${Metrics}_{nit}=\theta_{1}{Year2020}_{it}+\theta_{2}{Year2020}_{it}\times OneVehicle+\theta_{3}{Year2020}_{it}\times TwoVehicle+\theta_{4}{Year2020}_{it}\times ThreeVehicle+\theta_{5}{Year2020}_{it}\times FourMoreVehicle+\theta_{6}{Year2020}_{it}\times Transit+\gamma_{i}+\delta_{t} + u_{it}$ (A4)

Coefficient $\theta_{1}$ captures the relationship between the year and different metrics. Coefficient $\theta_{2}$, $\theta_{3}$, $\theta_{4}$, $\theta_{5}$, $\theta_{6}$ captures the additional effects of availability of one vehicle, availability of two vehicles, availability of three vehicles, availability of four or more vehicles, and the density of transit stops, respectively. Supplementary Table 3 shows the results of the DID estimation.

**Supplementary Table 3**. Mobility Disparities by Comparing 2019 and 2020

|  | (1)  Distance | (2)  Size | (3)  Percentage of Visitation |
| --- | --- | --- | --- |
| Order | 0.016***  (0.001) | 0.017***  (0.002) | 0.024***  (0.002) |
| Order× One vehicle available | 0.016***  (0.002) | 0.008**  (0.006) | 0.029***  (0.004) |
| Order× Two vehicles available | 0.014***  (0.003) | 0.012  (0.007) | 0.023***  (0.007) |
| Order× Three vehicles available | 0.018***  (0.002) | 0.002  (0.002) | 0.010***  (0.002) |
| Order× Four or more vehicles available | 0.002***  (0.0002) | 0.005  (0.0016) | 0.061***  (0.003) |
| Order× Density of transit stops | 0.007***  (0.002) | 0.002**  (0.001) | 0.035***  (0.001) |
| Census tract fixed effects | Yes | Yes | Yes |
| Date fixed effects | Yes | Yes | Yes |
| Observations | 1,686,908 | 1,686,908 | 1,686,908 |
| R-squared | 0.37 | 0.48 | 0.69 |

p<0.01 ***; p<0.05 **; p<0.1 *
